# Impact of renal function variability on long-term prognosis in ischemic stroke patients with atrial fibrillation

**DOI:** 10.3389/fneur.2024.1294022

**Published:** 2024-04-22

**Authors:** Xiao Wang, Chun-fung Sin, Kay-Cheong Teo, William C. Y. Leung, Yuen-Kwun Wong, Roxanna K. C. Liu, Joshua W. Fok, Bonaventure Y. Ip, Hon Hang Kwan, Tsz Ching Lee, Bun Sheng, Edwin Kin-Keung Yip, Desmond Y. H. Yap, Hao Luo, Kui-Kai Lau

**Affiliations:** ^1^Department of Medicine, School of Clinical Medicine, LKS Faculty of Medicine, The University of Hong Kong, Hong Kong, Hong Kong SAR, China; ^2^Department of Pathology, School of Clinical Medicine, LKS Faculty of Medicine, The University of Hong Kong, Hong Kong, Hong Kong SAR, China; ^3^Department of Medicine, Yan Chai Hospital, Hong Kong, Hong Kong SAR, China; ^4^Department of Medicine and Therapeutics, Faculty of Medicine, The Prince of Wales Hospital, The Chinese University of Hong Kong, Hong Kong, Hong Kong SAR, China; ^5^Department of Medicine and Geriatrics, Princess Margaret Hospital, Hong Kong, Hong Kong SAR, China; ^6^Department of Medicine and Geriatrics, Ruttonjee Hospital, Hong Kong, Hong Kong SAR, China; ^7^Department of Social Work and Social Administration, The University of Hong Kong, Hong Kong, Hong Kong SAR, China; ^8^Department of Computer Science, The University of Hong Kong, Hong Kong, Hong Kong SAR, China; ^9^The State Key Laboratory of Brain and Cognitive Sciences, The University of Hong Kong, Hong Kong, Hong Kong SAR, China

**Keywords:** renal function variability, ischemic stroke, atrial fibrillation, direct oral anticoagulant, warfarin

## Abstract

**Background:**

Although renal dysfunction is associated with adverse clinical outcomes in patients with atrial fibrillation (AF) following stroke, the impact of renal function variability is unclear.

**Aim:**

This study aimed to assess the association between renal function variability and various adverse clinical outcomes in patients with transient ischemic attack (TIA)/ischemic stroke and atrial fibrillation (AF).

**Methods:**

We conducted a population-based study and retrospectively identified patients hospitalized with a diagnosis of TIA/ischemic stroke and AF during 2016–2020 using the Clinical Data Analysis and Reporting System of Hong Kong. Serial serum creatinine tested upon the onset of TIA/ischemic stroke and during their subsequent follow-up was collected. Renal function variability was calculated using the coefficient of variation of the estimated glomerular filtration rate (eGFR). Clinical endpoints that occurred during the study period were captured and included ischemic stroke/systemic embolism, intracerebral hemorrhage (ICH), total bleeding, major adverse cardiovascular events (MACE), cardiovascular, non-cardiovascular, and all-cause mortality. Competing risk regression and Cox proportional hazard regression models were used to assess the associations of renal function variability with the outcomes of interest.

**Results:**

A total of 3,809 patients (mean age 80 ± 10 years, 43% men) who satisfied the inclusion and exclusion criteria were followed up for a mean of 2.5 ± 1.5 years (9,523 patient-years). The mean eGFR was 66 ± 22 mL/min/1.73 m^2^ at baseline, and the median number of renal function tests per patient during the follow-up period was 20 (interquartile range 11–35). After accounting for potential confounders, a greater eGFR variability was associated with increased risks of recurrent ischemic stroke/systemic embolism [fully adjusted subdistribution hazard ratio 1.11, 95% confidence interval (CI) 1.03–1.20], ICH (1.17, 1.01–1.36), total bleeding (1.13, 1.06–1.21), MACE (1.22, 1.15–1.30), cardiovascular (1.49, 1.32–1.69), non-cardiovascular (1.43, 1.35–1.52), and all-cause mortality (fully adjusted hazard ratio 1.44, 1.39–1.50).

**Conclusion:**

Visit-to-visit renal function variability is independently associated with adverse clinical outcomes in TIA/ischemic stroke patients with AF. Further large-scale studies are needed to validate our results.

## 1 Introduction

Oral anticoagulants (OACs), including warfarin and direct oral anticoagulants (DOACs), are crucial for primary and secondary prevention of thromboembolic events in patients with non-valvular atrial fibrillation (AF). DOACs have been shown in large randomized controlled trials to be at least as effective and even safer than warfarin and are now recommended by guidelines as first-line treatment in patients with non-valvular AF (1–6). All available DOACs, including dabigatran, rivaroxaban, apixaban, and edoxaban, are eliminated at least partly via the kidney (6, 7). Indications and dose adjustment of individual DOACs are also partially based on one’s renal function (6). Moreover, impaired renal function is reported to be associated with increased risks of adverse clinical outcomes in patients with AF (8–10).

Previous studies have largely investigated the clinical outcomes associated with renal function measured at a single time point, and the impact of renal function fluctuation has seldom been studied (8–10). Longitudinal intra-individual renal function variability is commonly observed in clinical practice, and high renal function variability is thought to be a result of poor kidney reserve and worsening autoregulatory ability (11). Studies have demonstrated that high renal function variability is associated with adverse clinical outcomes in a variety of conditions including hypertension, heart failure, and chronic kidney disease as well as in the general population (12–14). Renal function variability is also associated with an increased incidence of AF (15). Although a previous study evaluated the impact of renal function variability on the risk of major bleeding in patients with AF taking DOACs (16), the sample size was relatively small. The impact of renal function variability on other adverse clinical outcomes other than bleeding events remains uncertain (11).

We therefore aimed to assess the associations of renal function variability with a wide range of adverse clinical outcomes in patients with AF following TIA/ischemic stroke in a large population-based study.

## 2 Materials and methods

### 2.1 Data source

#### 2.1.1 Population-based cohort

We retrospectively identified and reviewed all TIA/ischemic stroke patients with AF who were acutely admitted to the public hospitals of Hong Kong (HK) due to their stroke during the period 1 January 2016 and 31 December 2020 using the Clinical Data Analysis and Reporting System (CDARS) of HK. CDARS is a territory-wide electronic healthcare database managed by the HK Hospital Authority. The Hospital Authority is the only public healthcare provider in HK and currently serves a population of more than 7 million people (17). The demographics, date of hospital admission and discharge, medical diagnosis, procedures, drug prescriptions, and results of laboratory tests of patients under the care of Hospital Authority are stored in CDARS. CDARS has been widely used in high-quality epidemiological research (18–20). The coding accuracy for diseases including stroke, AF, myocardial infarction, and gastrointestinal bleeding has been validated in previous studies with high positive and negative predictive values (18–20).

**Figure 1:**
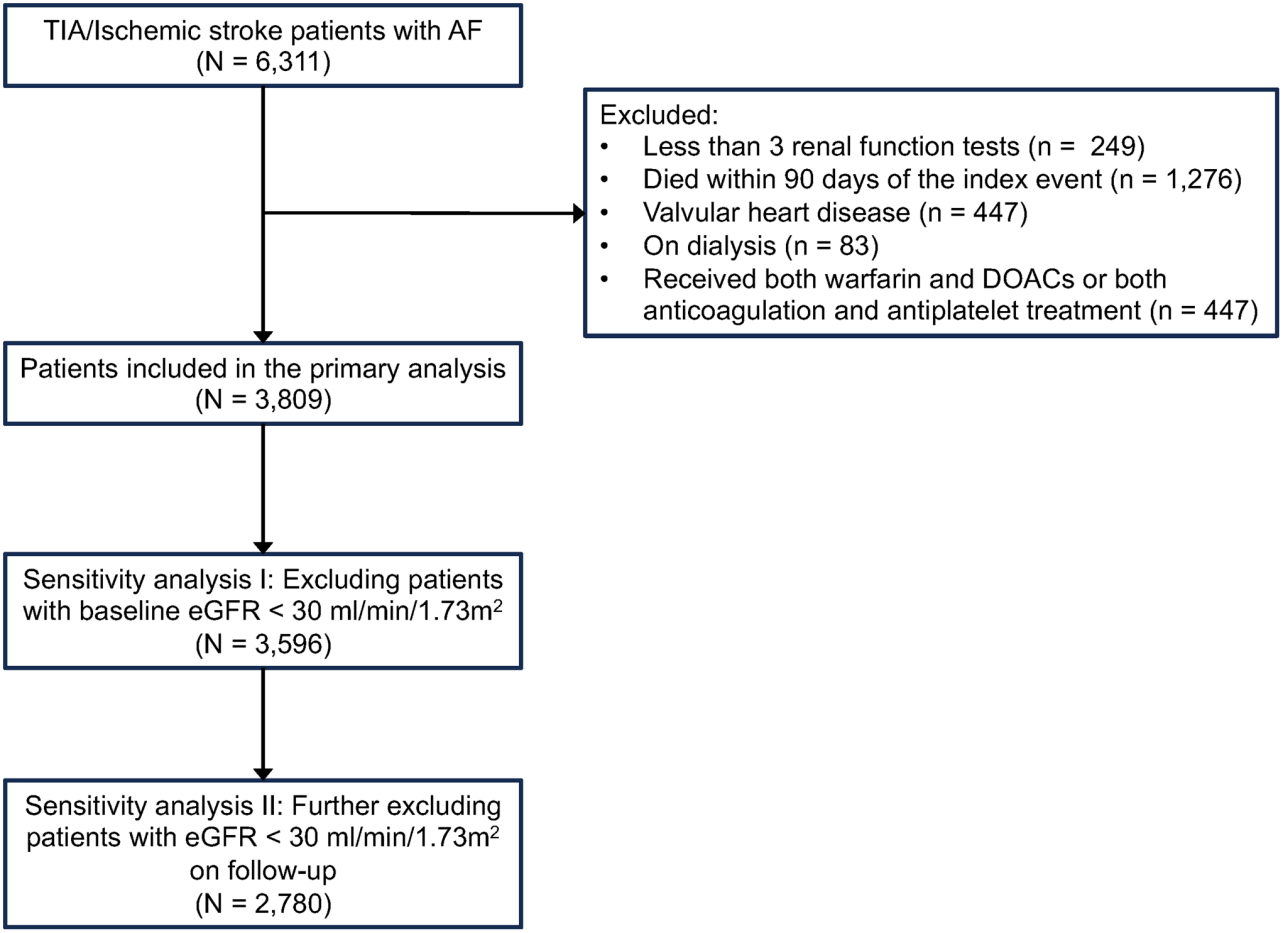
Flow of study patients.

Patients were identified using the following International Classification of Disease, Ninth Revision, Clinical Modification (ICD-9-CM) codes: TIA/ischemic stroke (433,434,435,436,437), AF (427.3). The index date was defined as the date of hospital admission due to TIA/ischemic stroke. Patients with a history of valvular replacement or valvular heart disease were excluded (ICD-9-CM; Supplementary Table 1). Patients who died within 90 days of the index date, received both warfarin and DOACs or both anticoagulation and antiplatelet therapy, had fewer than three renal function tests, and/or received dialysis during the study period were also excluded from the analysis (Figure 1).

We assessed several outcomes during the follow-up period: (1) ischemic stroke/systemic embolic events; (2) intracerebral hemorrhage (ICH); (3) total bleeding (intracranial and extracranial hemorrhage); (4) major adverse cardiovascular events (MACE); (5) all-cause mortality; (6) cardiovascular mortality; and (7) non-cardiovascular mortality. MACE was defined as a composite of recurrent stroke, acute coronary syndrome, new-onset peripheral vascular disease, congestive heart failure warranting hospitalization, and cardiovascular mortality. Cardiovascular mortality was defined as death within 30 days following stroke, acute coronary syndrome, or congestive heart failure. The ICD-9-CM codes used to identify outcomes of interest are listed in Supplementary Table 1. Patients were followed up until the outcomes of interest, death, or 30 June 2021, whichever occurred earlier.

All records of medical diagnosis dated before the index date were retrieved using the ICD-9-CM codes. Baseline medical conditions including hypertension, diabetes mellitus, hyperlipidemia, prior TIA/stroke, and prior ischemic heart disease were assessed based on these coded medical diagnoses. The details of ICD-9 codes used are listed in Supplementary Table 1.

#### 2.1.2 Hospital-based stroke registries

To enable a more accurate adjudication of outcome events and validation of findings from a population-based cohort of patients identified from electronic health records, we further studied patients with AF following TIA/ischemic stroke from several existing hospital-based stroke registries in HK. These included stroke patients who were admitted to the acute stroke units of Queen Mary Hospital from June 2013 to December 2020, and stroke patients who were admitted to the acute stroke units of Princess Margaret, Yan Chai, and Ruttonjee Hospitals from September 2019 to December 2020.

Using these stroke registries, we retrospectively identified patients with: (1) acute ischemic stroke [defined as acute-onset focal neurological deficits with corresponding lesion on diffusion weighted (DWI) magnetic resonance imaging (MRI) or computed tomography (CT)] or TIA (defined as acute-onset focal neurological deficits of presumed ischemic origin without a corresponding lesion on DWI or if no MRI was acquired, lasting less than 24 h); (2) AF, confirmed by electrocardiogram (ECG) or prolonged rhythm monitoring, either known before the index event or detected during admission; (3) more than three renal function tests performed during follow-up; (4) survival longer than 90 days after the index event; (5) without significant valvular heart disease or previous valvular replacement; (6) not undergoing dialysis; and (7) not on both antiplatelet and anticoagulation treatment.

All patients in the stroke registries were followed up by a clinician every 3 to 6 months, or more frequently if clinically indicated. We retrieved patient’s demographic data, past medical history, details of the index event, medications upon hospital discharge, and serial renal function test results during the follow-up period from these hospital-based stroke registries and supplemented these from the Hospital Authority electronic health records where necessary. Follow-up outcomes were assessed independently by two experienced neurologists after reviewing patients’ electronic health records. We assessed the following outcomes: (1) ischemic stroke/systemic embolic events, (2) ICH, (3) major bleeding, (4) MACE, (5) all-cause mortality, (6) cardiovascular mortality, and (7) non-cardiovascular mortality. Patients were followed up until the outcomes of interest, death, or 30 June 2021, whichever occurred earlier.

Recurrent stroke was defined as the sudden onset of new neurological symptoms fitting the definition of ICH or ischemic stroke following a neurologically stable period and not attributable to hemorrhagic transformation, mass effect, or cerebral edema. CT or MRI was used to support the diagnosis if recurrent stroke was suspected. A bleeding episode was defined as major if it was fatal or associated with at least one of the following criteria: (i) a fall in hemoglobin level by ≥2 g/dL or documented transfusion of at least two units of red blood cells; (ii) life-threatening bleeding; and/or (iii) involvement of a critical anatomical site (intracranial, spinal, ocular, pericardial, articular, intramuscular with compartment syndrome, or retroperitoneal) (21).

The study protocol was approved by the Institutional Review Board of the University of Hong Kong/Hospital Authority Hong Kong West Cluster (reference number UW21-463/UW18-361). Informed consent was not required in the population-based study or for the retrospective review of patients from pre-existing hospital-based stroke registries.

### 2.2 Calculation of renal function variability

The results of serial renal function tests between the onset of the TIA/ischemic stroke and the occurrence of outcomes of interest were extracted from CDARS. The glomerular filtration rate (GFR) was estimated by using a modified 4-variable Chronic Kidney Disease Epidemiology Collaboration (CKD-EPI) formula with an adjusted coefficient of 1.049 for Asian populations (22). The formula is as follows:


$$\begin{array}{l} Estimated\;GFR\;{\left(eGFR\right)}_{CKD}{\;}_{-EPI}=141\times \min\;\left(\frac{SCr}{\kappa },1\right)\alpha \times \max\left(\frac{SCr}{\kappa },1\right)\\ \;\;\;\;\;\;\;\;\;\;\;\;\;\;\;\;\;\;\;\;\;\;\;\;\;\;\;-1.209\times 0.993Age\times 1.018\left(iffemeale\right)\times 1.049 \end{array}$$

where SCr was serum creatinine, κ was 0.7 for women and 0.9 for men, α was − 0.329 for women and −0.411 for men, “min” was the minimum of SCr/κ or 1, and “max” indicated the maximum of SCr/κ or 1 (22). For each patient, renal function variability was defined as the coefficient of variation (CV) [standard deviation (SD) divided by the mean eGFR] (12). Renal function variability was calculated from the results of serial renal function tests between the onset of the TIA/ischemic stroke and the occurrence of outcomes of interest.

### 2.3 Statistical analysis

Patients were divided into four groups (Q1–Q4) based on their eGFR CV quartiles for each outcome of interest. Continuous variables were presented as mean (SD), and analysis of variance was used to compare the differences between groups. For data not normally distributed, the median value (interquartile range) was presented, and the Kruskal–Wallis test was used for comparison between groups. Categorical variables were presented as numbers (proportions). A chi-squared test or Fisher’s exact test was used to compare differences between the groups where appropriate.

The Kaplan–Meier survival analysis was used to examine the cumulative event rate of outcomes of interest among patients in each group with different renal function variability, and the cumulative event rates of each group were compared using a log-rank test. To assess the association of renal function variability with all-cause mortality, Cox proportional hazard regression was performed to compute the hazard ratio (HR) with a 95% confidence interval (CI). To determine the associations of renal function variability with outcomes other than all-cause mortality, competing risk regression was conducted to estimate the subdistribution hazard ratios (SHRs) by considering death as a competing event according to the method of Fine and Gray (23). Three models were constructed for each outcome of interest: a univariate model, a model adjusted for age and sex, and a multivariable model adjusting for all potential confounders including age, sex, baseline estimated glomerular filtration rate (eGFR), hypertension, diabetes mellitus, hyperlipidemia, history of TIA/stroke, history of ischemic heart disease, prescription of angiotensin-converting enzyme inhibitors (ACEIs), angiotensin receptor blockers (ARBs), and statins. Variables with a *p*-value of <0.1 upon univariate analyses were entered into a multivariate model for analyses. Interaction analysis was also performed to determine whether there was an interaction between eGFR variability and the types of antithrombotic treatment for each clinical outcome.

To take into account of potential non-linearity between renal function variability and clinical outcomes, we employed a restricted cubic spline to perform regression. A total of four knots were utilized in multivariable-adjusted analyses.

We performed further sensitivity analyses by, first of all, repeating the main analyses after excluding patients with eGFR < 30 mL/min/1.73 m^2^ at baseline. To ensure that the associations of renal function variability with adverse clinical endpoints were not driven by a significant decline in eGFR during follow-up, a second sensitivity analysis was performed with further exclusion of patients who had an eGFR < 30 mL/min/1.73 m^2^ at any time point during the follow-up period (Figure 1). To ensure that the associations between renal function variability and clinical outcomes were not reliant on the specific measurement of renal function variability, we also performed similar analyses using alternative methods for assessing renal function variability, including variability independent of mean (VIM) (15, 24) and average real variability (ARV) (25). A *p*-value of 0.05 was considered statistically significant.

All statistical analyses were carried out using R (version 4.3.2) and RStudio version 1.3.1093 (RStudio, PBC 2020).

## 3 Results

### 3.1 Population-based study

#### 3.1.1 Baseline characteristics

A total of 6,311 patients with TIA/ischemic stroke and AF were identified from CDARS during the study period. After excluding patients with fewer than three renal function tests during the follow-up period, patients who died within 90 days of the index stroke, were undergoing dialysis, had underlying valvular heart disease/undergone valvular replacement, and/or received both warfarin and DOACs or both antiplatelets and anticoagulants during the study period, a total of 3,809 patients were included in the final analysis (Figure 1).

Baseline characteristics of patients recruited during the study period are shown in Table 1. The mean age of the study population was 80 ± 10 years, and 43% were male with a mean eGFR of 66 ± 22 mL/min/1.73 m^2^ at baseline. The median number of renal function tests performed in each patient was 20 (interquartile range 11–35). Patients with a higher renal function variability were older, were more likely to be female, had a lower baseline eGFR, and were more likely to have a higher burden of vascular risk factors and comorbidities (all *p* < 0.01; Table 1). Patients with greater renal function variability were also more likely to be prescribed antiplatelet agents or no antithrombotic agents upon discharge (all *p* < 0.05; Table 1).

**Table 1:** Baseline characteristics of study participants and their outcomes by quartiles of eGFR variability.

	All	Quartile				*p*-value
	(*n* = 3,809)	1 (*n* = 953)	2 (*n* = 952)	3 (*n* = 952)	4 (*n* = 952)	
Baseline clinical characteristics^*^						
Age, y (SD)	80.1 (10.4)	75.3 (11.3)	79.4 (10.6)	82.3 (9.4)	83.3 (8.4)	<0.001
Male sex, (%)	1,637 (43.0)	472 (49.5)	450 (47.3)	385 (40.4)	330 (34.7)	<0.001
eGFR, ml/min/1.73m^2^ (SD)	66.2 (22.0)	82.1 (17.7)	68.6 (18.1)	60.4 (19.7)	53.5 (21.4)	<0.001
No. of renal function tests [IQR]	20 [11-35]	12 [7-19]	17 [10-27]	25 [15-37]	32 [19-49]	<0.001
CV† of eGFR (SD)	0.18 (0.12)	0.07 (0.02)	0.13 (0.02)	0.19 (0.02)	0.34 (0.11)	<0.001
CHA_2_DS_2_-VASc score [IQR]	4 [3-5]	3 [2-4]	4 [3-5]	4[3-5]	5 [3-6]	<0.001
Medical history						
Hypertension (%)	2,341 (61.5)	452 (47.4)	565 (59.3)	632 (66.4)	692 (72.7)	<0.001
Diabetes mellitus (%)	1,002 (26.3)	177 (18.6)	228 (23.9)	270 (28.4)	327 (34.3)	<0.001
Hyperlipidemia (%)	864 (22.7)	189 (19.8)	199 (20.9)	240 (25.2)	236 (24.8)	0.007
Prior TIA/stroke (%)	1,036 (27.2)	207 (21.7)	265 (27.8)	278 (29.2)	286 (30.0)	<0.001
Ischemic heart disease (%)	1,604 (42.1)	262 (27.5)	374 (39.3)	465 (48.8)	503 (52.8)	<0.001
Antithrombotic drugs at discharge						
Antiplatelet (%)	1,365 (35.8)	229 (24.0)	292 (30.7)	414 (43.5)	430 (45.2)	<0.001
DOAC (%)	1738 (45.6)	559 (58.7)	494 (51.9)	370 (38.9)	315 (33.1)	<0.001
Apixaban (%)	833 (21.9)	231 (24.2)	237 (24.9)	189 (19.9)	176 (18.5)	<0.001
Dabigatran (%)	594 (15.6)	241 (25.3)	169 (17.8)	108 (11.3)	76 (7.98)	<0.001
Edoxaban (%)	37 (1.0)	11 (1.2)	13 (1.4)	9 (1.0)	4 (0.4)	0.030
Rivaroxaban (%)	274 (7.2)	76 (8.0)	75 (7.9)	64 (6.7)	59 (6.2)	0.370
Warfarin (%)	418 (11.0)	102 (10.7)	103 (10.8)	98 (10.3)	115 (12.1)	0.629
No antithrombotic drugs (%)	288 (7.6)	63 (6.6)	63 (6.6)	70 (7.4)	92 (9.7)	0.036
Concomitant drug use						
ACEIs/ARBs (%)	1,128 (29.6)	228 (23.9)	280 (29.4)	301 (31.6)	319 (33.5)	<0.001
Statins (%)	2,642 (69.4)	677 (71.0)	676 (71.0)	630 (66.2)	659 (69.2)	0.194
Outcomes						
Recurrent stroke (%)	453 (11.9)	73 (7.7)	107 (11.2)	124 (13.0)	149 (15.7)	<0.001
Ischemic stroke (%)	368 (9.7)	58 (6.1)	85 (8.9)	108 (11.3)	117 (12.3)	<0.001
Intracerebral hemorrhage (%)	93 (2.4)	16 (1.7)	24 (2.5)	17 (1.8)	36 (3.8)	0.010
Systemic embolism (%)	22 (0.6)	0 (0.0)	6 (0.6)	5 (0.5)	11 (1.2)	0.011
Acute coronary event (%)	98 (2.6)	7 (0.7)	16 (1.7)	28 (2.9)	47 (4.9)	<0.001
Extracranial bleeding (%)	582 (15.3)	103 (10.8)	130 (13.7)	169 (17.8)	180 (18.9)	<0.001
Gastrointestinal bleeding (%)	409 (10.7)	76 (8.0)	92 (9.7)	121 (12.7)	120 (12.6)	0.001
Other bleeding (%)	205 (5.4)	33 (3.5)	47 (4.9)	58 (6.1)	67 (7.0)	0.004
Major adverse cardiovascular event (%)	992 (26.0)	114 (12.0)	211 (22.2)	291 (30.6)	376 (39.5)	<0.001
Death (%)	1,562 (41.0)	154 (16.2)	251 (26.4)	471 (49.5)	686 (72.1)	<0.001

^*^Data for continuous variables are expressed as mean (SD) or median [IQR] as appropriate. ^†^Coefficient of variation. eGFR, estimated glomerular filtration rate; SD, standard deviation; IQR, interquartile range; TIA, transient ischemic attack; DOAC, direct oral anticoagulants; ACEIs, angiotensin-converting enzyme inhibitors; ARBs, angiotensin receptor blockers.

During a mean follow-up of 2.5 ± 1.5 years (9,523 patient-years), 368 (9.7%) ischemic strokes, 22 (0.6%) systemic embolic events, 93 (2.4%) ICH, 582 (15.3%) extracranial bleeding events, and 992 (26.0%) MACEs occurred within the study population. A total of 1,562 (41.0%) patients died during the follow-up period (Table 1).

#### 3.1.2 Renal function variability and clinical outcomes

**Figure 2:**
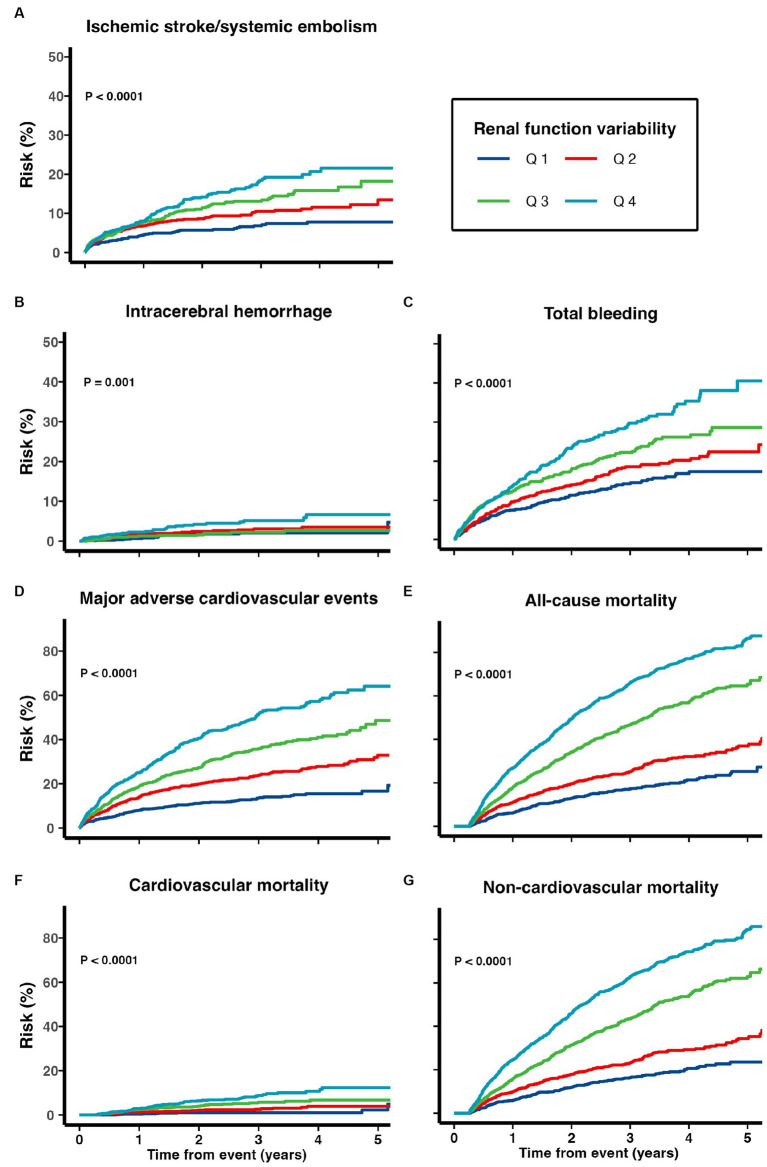
Risk of **(A)** recurrent ischemic stroke and systemic embolism, **(B)** intracerebral hemorrhage, **(C)** total bleeding, **(D)** major adverse cardiovascular events, **(E)** all-cause mortality, **(F)** cardiovascular mortality, and **(G)** non-cardiovascular mortality among patients with TIA/ischemic stroke and AF.

Patients with higher renal function variability were at increased risk of all adverse clinical outcomes including ischemic stroke/systemic embolism, ICH, total bleeding, MACE, cardiovascular, non-cardiovascular and all-cause mortality (all p_trend_ < 0.05; Table 2; Figure 2). When treated as a continuous variable, greater eGFR variability (expressed as per 1 SD greater in CV) was associated with increased risk of ischemic stroke/systemic embolism (multivariate-adjusted SHR 1.11, 95% CI 1.03–1.20), ICH (1.17, 1.01–1.36), total bleeding (1.13, 1.06–1.21), MACE (1.22, 1.15–1.30), as well as all-cause (multivariate-adjusted HR 1.44, 1.39–1.50), cardiovascular (1.49, 1.32–1.69), and non-cardiovascular mortality (1.43, 1.35–1.52; all *p* < 0.05; Figure 2; Table 3).

**Table 2:** Competing risk regression analysis by the quartiles of eGFR variability.

Coefficient of variance of eGFR	Event	Follow-up duration (person-years)	Incidence rate (per 1,000 person-years)	Unadjusted SHR (95% CI)	*P*_trend_	SHR (95% CI) adjusted for age and sex	*P*_trend_	SHR (95% CI) adjusted for age sex and vascular risk factors^*^	*P*_trend_
Ischemic stroke and systemic embolism									
Q1	58	2338.2	24.8	1	<0.001	1	<0.001	1	<0.001
Q2	91	2361.7	38.5	1.47 (1.10–1.98)		1.51 (1.13–2.03)		1.49 (1.11–2.00)	
Q3	113	2076.9	54.4	1.78 (1.34–2.36)		1.85 (1.39–2.47)		1.80 (1.34–2.41)	
Q4	128	1793.9	71.3	1.81 (1.36–2.39)		1.88 (1.41–2.50)		1.81 (1.35–2.42)	
Intracerebral hemorrhage									
Q1	16	2443.5	6.5	1	0.011	1	0.005	1	0.020
Q2	24	2464.3	9.7	1.76 (0.97–3.18)		1.80 (0.99–3.29)		1.68 (0.92–3.05)	
Q3	17	2220.9	7.7	1.26 (0.67–2.36)		1.36 (0.71–2.62)		1.24 (0.64–2.38)	
Q4	36	1917.5	18.8	2.33 (1.33–4.10)		2.62 (1.43–4.77)		2.28 (1.24–4.17)	
Total bleeding									
Q1	118	2268.1	52.0	1	<0.001	1	<0.001	1	<0.001
Q2	150	2252.2	66.6	1.30 (1.04–1.63)		1.23 (0.98–1.55)		1.25 (0.99–1.58)	
Q3	182	1972.3	92.3	1.49 (1.20–1.86)		1.39 (1.10–1.75)		1.43 (1.13–1.81)	
Q4	212	1690.1	125.4	1.76 (1.42–2.17)		1.64 (1.31–2.06)		1.71 (1.35–2.19)	
Major adverse cardiovascular events									
Q1	114	2260.7	50.4	1	<0.001	1	<0.001	1	<0.001
Q2	211	2187.3	96.5	1.87 (1.52–2.30)		1.84 (1.50–2.27)		1.78 (1.44–2.20)	
Q3	291	1854.9	156.9	2.66 (2.19–3.24)		2.57 (2.10–3.14)		2.38 (1.94–2.93)	
Q4	376	1467.0	256.3	3.53 (2.91–4.27)		3.37 (2.77–4.10)		3.10 (2.52–3.81)	
All-cause mortality^†^									
Q1	154	2457.4	62.7	1	<0.001	1	<0.001	1	<0.001
Q2	251	2495.3	100.6	1.60 (1.32–1.93)		1.27 (1.04–1.53)		1.30 (1.07–1.58)	
Q3	471	2244.4	209.9	3.36 (2.82–4.00)		2.28 (1.91–2.72)		2.22 (1.85–2.66)	
Q4	686	1965.1	349.1	5.61 (4.75–6.63)		3.70 (3.12–4.39)		3.64 (3.03–4.36)	
Cardiovascular mortality									
Q1	10	2457.4	4.1	1	<0.001	1	<0.001	1	<0.001
Q2	23	2495.3	9.2	2.16 (1.06–4.43)		1.84 (0.89–3.78)		1.87 (0.91–3.83)	
Q3	41	2244.4	18.3	4.22 (2.18–8.18)		3.16 (1.62–6.17)		3.09 (1.57–6.09)	
Q4	58	1965.1	29.5	7.67 (4.06–14.49)		5.55 (2.91–10.57)		5.34 (2.74–10.41)	
Non-cardiovascular mortality									
Q1	144	2457.4	58.6	1	<0.001	1	<0.001	1	<0.001
Q2	228	2495.3	91.4	1.56 (1.26–1.93)		1.22 (0.99–1.50)		1.25 (1.01–1.54)	
Q3	430	2244.4	191.6	3.29 (2.72–3.98)		2.23 (1.83–2.70)		2.14 (1.76–2.61)	
Q4	628	1965.1	319.6	5.54 (4.62–6.65)		3.60 (2.98–4.34)		3.50 (2.87–4.26)	

^*^Risk factors include hypertension, dyslipidemia, diabetes mellitus, history of TIA/stroke, history of ischemic heart disease, eGFR at baseline, antithrombotic treatment, ACEIs, ARBs, statins. Risk factors with a *p*-value of <0.1 in the univariate model are included in the final model. ^†^Hazard ratio; SHR, subdistribution hazard ratio; CI, confidence interval; eGFR, estimated glomerular filtration rate; TIA, transient ischemic attack; ACEIs, angiotensin-converting enzyme inhibitors; ARBs, angiotensin receptor blockers.

**Table 3:** Competing risk regression analysis by renal function variability.

	Renal function variability as an interval variable (per 1 SD increment in CV)					
	Unadjusted SHR (95% CI)	*P*-value	SHR (95% CI) adjusted for age and sex	*P*-value	Multivariate^*^ adjusted SHR (95% CI)	*P*-value
Ischemic stroke and systemic embolism	1.16 (1.07–1.24)	<0.001	1.15 (1.07–1.24)	0.002	1.11(1.03–1.20)	0.009
Intracerebral hemorrhage	1.19 (1.03–1.37)	0.019	1.22 (1.05–1.41)	0.008	1.17 (1.01–1.36)	0.041
Total bleeding	1.14 (1.08–1.21)	<0.001	1.12 (1.06–1.20)	0.001	1.13 (1.06–1.21)	<0.001
Major adverse cardiovascular events	1.30 (1.22–1.37)	<0.001	1.28 (1.21–1.35)	<0.001	1.22 (1.15–1.30)	<0.001
All-cause mortality^†^	1.53 (1.48–1.58)	<0.001	1.49 (1.44–1.55)	<0.001	1.44 (1.39–1.50)	<0.001
Cardiovascular mortality	1.57 (1.39–1.76)	<0.001	1.54 (1.38–1.73)	<0.001	1.49 (1.32–1.69)	<0.001
Non-cardiovascular mortality	1.53 (1.42–1.64)	<0.001	1.49 (1.41–1.57)	<0.001	1.43 (1.35–1.52)	<0.001

^*^Covariates included age, sex, hypertension, hyperlipidemia, diabetes mellitus, history of TIA/stroke, history of ischemic heart disease, eGFR at baseline, antithrombotic treatment, angiotensin converting enzyme inhibitor, angiotensin receptor blocker and statin-use. Covariates with a *p*-value of <0.1 in the univariate model were included in the final model. ^†^Hazard ratio. SD, standard deviation; CV, coefficient of variation; SHR, subdistribution hazard ratio; CI, confidence interval; eGFR, estimated glomerular filtration rate; TIA, transient ischemic attack.

Supplementary Figure 1 depicts the association between renal function variability expressed in the CV of eGFR and adverse clinical outcomes using restricted cubic splines analysis. Non-linear association of eGFR CV with ischemic stroke/systemic embolism, total bleeding, MACE, all-cause, and non-cardiovascular mortality was observed (all *p* for non-linear < 0.05; Supplementary Figure 1). The association of various clinical outcomes and renal function variability, estimated using the restricted cubic splines analysis, aligned with the findings from competing risk regression. The risk for ischemic stroke/systemic embolism increased at lower eGFR CV levels, reaching a plateau at approximately 0.3 before a gradual decrease afterward. The risk for total bleeding and MACE increased rapidly when the eGFR CV was relatively low and then reached a plateau at approximately 0.3 (Supplementary Figure 1).

Significant interactions between eGFR variability and the types of antithrombotic treatment in association with ischemic stroke/systemic embolism (*p* for interaction = 0.033), total bleeding (p for interaction = 0.005), all-cause mortality (*p* for interaction < 0.001), and non-cardiovascular mortality (*p* for interaction < 0.001) were also noted (Table 4).

**Table 4:** Competing risk regression analysis by renal function variability stratified by antithrombotic strategies.

	Renal function variability as an interval variable (per 1 SD increment in CV)						
	Unadjusted SHR (95% CI)	*P*-value	SHR (95% CI) adjusted for age and sex	*P*-value	Multivariate^*^ adjusted SHR (95% CI)	*P*-value	*P* for interaction
Ischemic stroke and systemic embolism							
DOAC users (*n* = 1,738)	1.32 (1.10–1.58)	0.003	1.38 (1.16–1.65)	<0.001	1.38 (1.16–1.65)	<0.001	0.033
Warfarin users (*n* = 418)	1.24 (1.01–1.51)	0.037	1.19 (0.94–1.49)	0.140	1.18 (0.95–1.48)	0.130	
Antiplatelet users (*n* = 1,365)	0.98 (0.87–1.11)	0.790	0.99 (0.88–1.12)	0.860	0.98 (0.86–1.11)	0.720	
Intracerebral hemorrhage							
DOAC users (*n* = 1,738)	1.41 (1.08–1.84)	0.022	1.40 (1.03–1.91)	0.034	1.40 (1.03–1.91)	0.034	0.693
Warfarin users (*n* = 418)	1.15 (0.85–1.54)	0.370	1.18 (0.89–1.56)	0.260	1.19 (0.90–1.57)	0.230	
Antiplatelet users (*n* = 1,365)	1.15 (0.90–1.48)	0.260	1.17 (0.93–1.49)	0.190	1.13 (0.88–1.44)	0.340	
Total bleeding							
DOAC users (*n* = 1,738)	1.24 (1.10–1.40)	<0.001	1.23 (1.07–1.41)	0.003	1.23 (1.07–1.41)	0.003	0.005
Warfarin users (*n* = 418)	1.16 (1.00–1.34)	0.053	1.13 (0.97–1.32)	0.110	1.12 (0.95–1.31)	0.180	
Antiplatelet users (*n* = 1,365)	1.00 (0.90–1.12)	0.990	1.01 (0.90–1.13)	0.920	1.01 (0.90–1.13)	0.920	
Major adverse cardiovascular events							
DOAC users (*n* = 1,738)	1.31 (1.20–1.44)	<0.001	1.29 (1.17–1.42)	<0.001	1.26 (1.14–1.40)	<0.001	0.129
Warfarin users (*n* = 418)	1.33 (1.19–1.49)	<0.001	1.31 (1.16–1.46)	<0.001	1.25 (1.11–1.41)	<0.001	
Antiplatelet users (*n* = 1,365)	1.22 (1.12–1.33)	<0.001	1.21 (1.11–1.32)	<0.001	1.18 (1.08–1.29)	<0.001	
All-cause mortality^†^							
DOAC users (*n* = 1,738)	1.75 (1.62–1.90)	<0.001	1.77 (1.63–1.93)	<0.001	1.79 (1.64–1.95)	<0.001	<0.001
Warfarin users (*n* = 418)	1.61 (1.47–1.75)	<0.001	1.51 (1.38–1.66)	<0.001	1.52 (1.37–1.67)	<0.001	
Antiplatelet users (*n* = 1,365)	1.32 (1.26–1.39)	<0.001	1.31 (1.24–1.39)	<0.001	1.29 (1.21–1.38)	<0.001	
Cardiovascular mortality							
DOAC users (*n* = 1,738)	1.87 (1.49–2.34)	<0.001	1.82 (1.47–2.25)	<0.001	1.74 (1.35–2.24)	<0.001	0.302
Warfarin users (*n* = 418)	1.48 (1.16–1.90)	<0.001	1.43 (1.10–1.86)	0.008	1.48 (1.14–1.92)	0.003	
Antiplatelet users (*n* = 1,365)	1.43 (1.22–1.68)	<0.001	1.45 (1.23–1.72)	<0.001	1.43 (1.20–1.71)	<0.001	
Non-cardiovascular mortality							
DOAC users (*n* = 1,738)	1.74 (1.54–1.97)	<0.001	1.76 (1.6–1.95)	<0.001	1.78 (1.61–1.98)	<0.001	<0.001
Warfarin users (*n* = 418)	1.62 (1.43–1.84)	<0.001	1.53 (1.32–1.76)	<0.001	1.56 (1.35–1.81)	<0.001	
Antiplatelet users (*n* = 1,365)	1.31 (1.22–1.40)	<0.001	1.30 (1.21–1.38)	<0.001	1.28 (1.20–1.37)	<0.001	

^*^Covariates included age, sex, hypertension, hyperlipidemia, diabetes mellitus, history of TIA/stroke, history of ischemic heart disease, eGFR at baseline, angiotensin-converting enzyme inhibitor, angiotensin receptor blocker and statin-use. Covariates with a *p*-value of <0.1 in the univariate model were included in the final model. ^†^Hazard ratio. SD, standard deviation; CV, coefficient of variation; SHR, subdistribution hazard ratio; CI, confidence interval; eGFR, estimated glomerular filtration rate; TIA, transient ischemic attack; DOAC, direct oral anticoagulants.

#### 3.1.3 DOAC users

**Figure 3:**
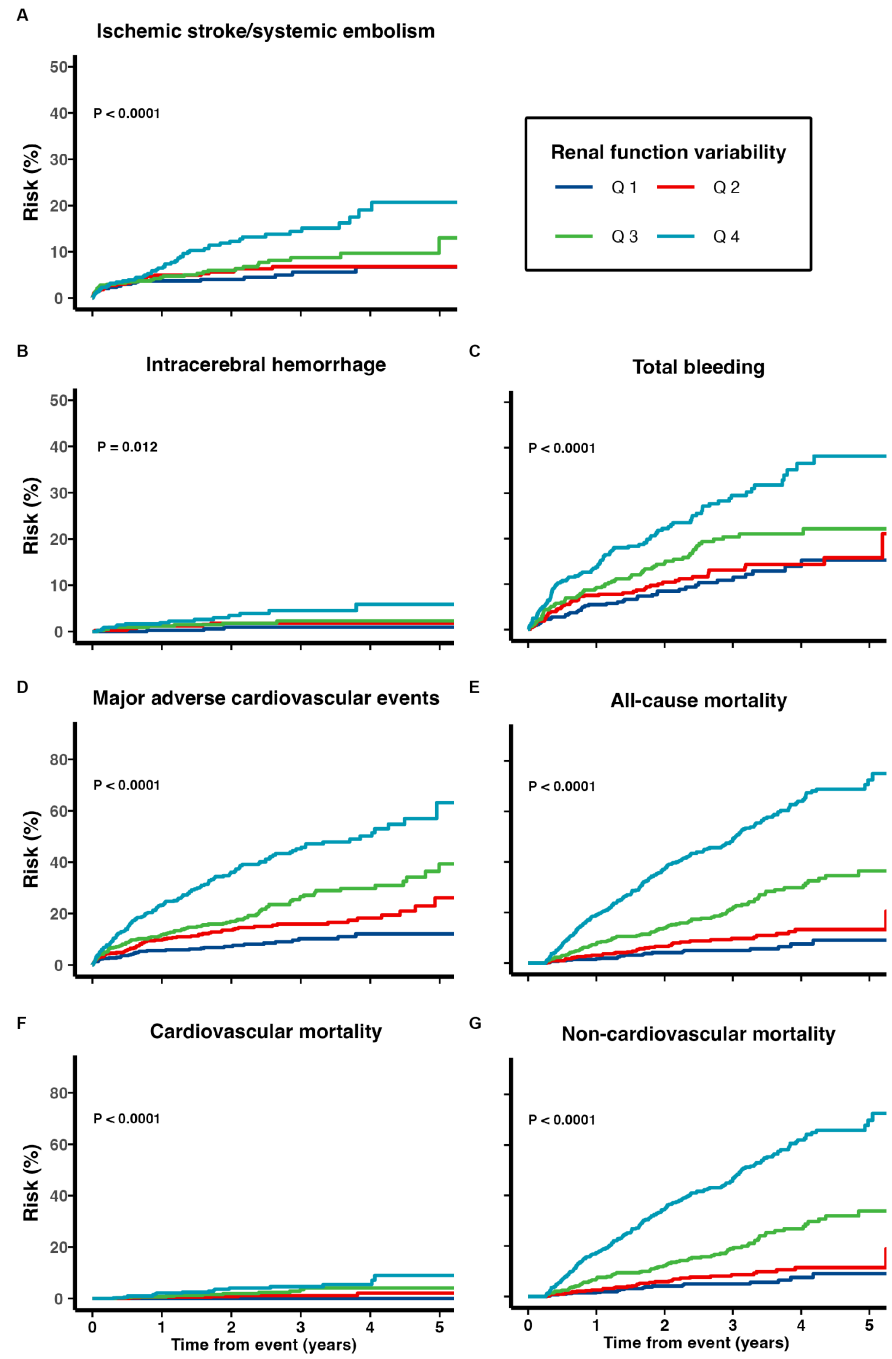
Risk of **(A)** recurrent ischemic stroke and systemic embolism, **(B)** intracerebral hemorrhage, **(C)** total bleeding, **(D)** major adverse cardiovascular events, **(E)** all-cause mortality, **(F)** cardiovascular mortality, and **(G)** non-cardiovascular mortality among patients with TIA/ischemic stroke and AF on DOACs.

Cumulative risks of various adverse outcomes are shown in Figure 3 for the 1,738 patients prescribed with DOACs on discharge (Figure 3). When treated as a continuous variable, greater eGFR variability was associated with increased risk of recurrent ischemic stroke/systemic embolism (multivariate-adjusted SHR 1.38, 1.16–1.65), ICH (1.40, 1.03–1.91), total bleeding (1.23, 1.07–1.41), MACE (1.26, 1.14–1.40), all-cause (1.79, 1.64–1.95), cardiovascular (1.74, 1.35–2.24), and non-cardiovascular mortality (1.78, 1.61–1.98; all *p* < 0.05; Table 4).

When stratified by individual DOACs [no further analysis was performed in edoxaban users due to the small sample size (*n* = 37)], greater eGFR variability was associated with increased risks of MACE, all-cause, and non-cardiovascular mortality in all three DOACs (apixaban, dabigatran, and rivaroxaban; Supplementary Table 2). Higher renal function variability was also significantly associated with recurrent ischemic stroke/systemic embolism and total bleeding events in dabigatran and apixaban users, as well as ICH and cardiovascular mortality among apixaban users (Supplementary Table 2).

#### 3.1.4 Warfarin users

**Figure 4:**
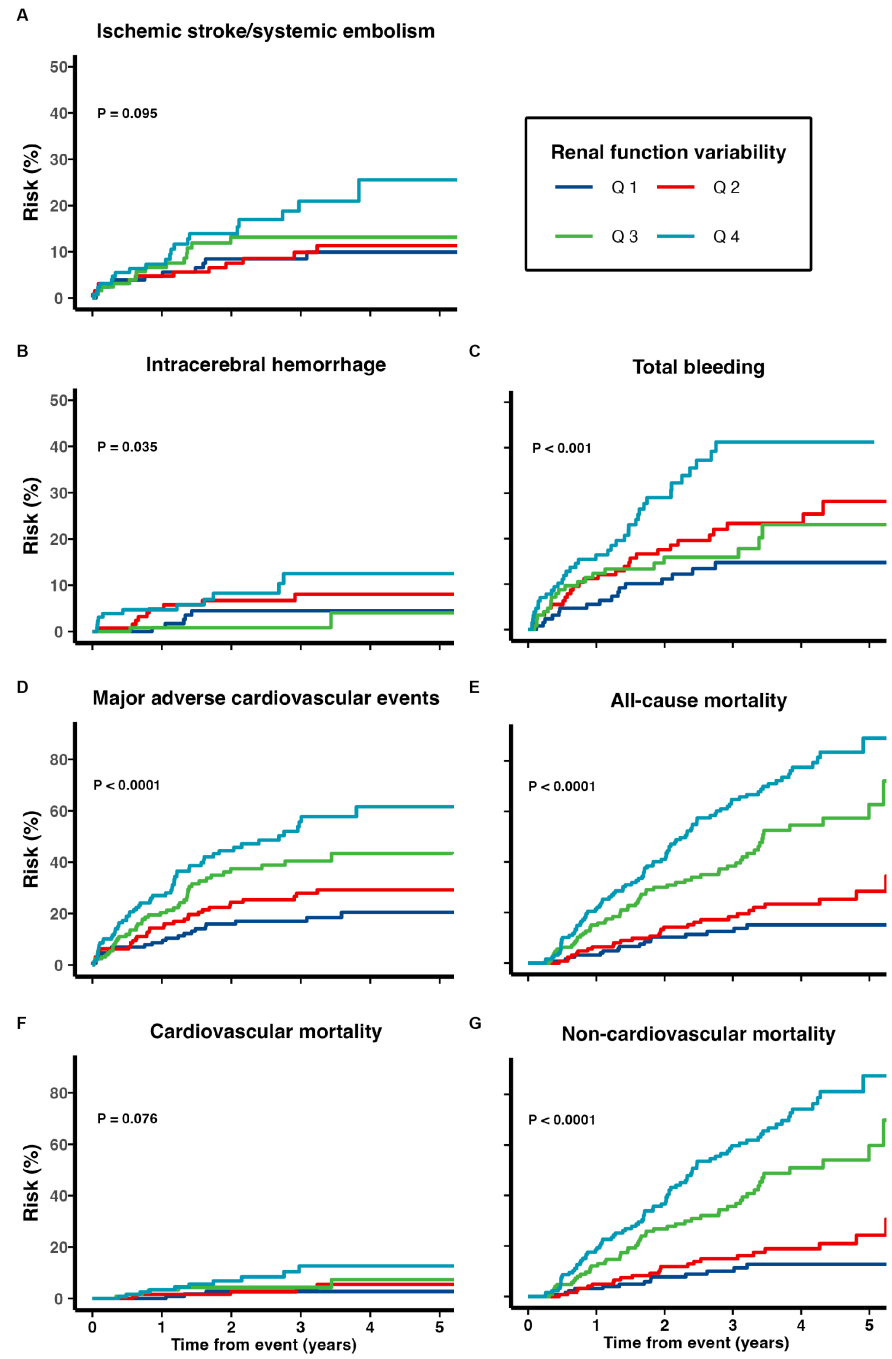
Risk of **(A)** recurrent ischemic stroke and systemic embolism, **(B)** intracerebral hemorrhage, **(C)** total bleeding, **(D)** major adverse cardiovascular events, **(E)** all-cause mortality, **(F)** cardiovascular mortality, and **(G)** non-cardiovascular mortality among patients with TIA/ischemic stroke and AF on warfarin.

The cumulative risks of various adverse outcomes are shown in Figure 4 for the 418 patients discharged with warfarin. A greater renal function variability was significantly associated with subsequent risks of MACE (multivariate-adjusted SHR 1.25, 95% CI 1.11–1.41), all-cause (1.52, 1.37–1.67), cardiovascular (1.48, 1.14–1.92), and non-cardiovascular mortality (1.56, 1.35–1.81; all *p* < 0.01), while the risks of recurrent ischemic stroke/systemic embolism, ICH, and total bleeding events were not increased in patients with a greater renal function variability after adjusting for confounding variables (all *p* > 0.05; Table 4).

#### 3.1.5 Antiplatelet users

A total of 1,365 patients were discharged with antiplatelet drugs only. A greater renal function variability was significantly associated with an increased risk of MACE (multivariate-adjusted SHR 1.18, 95% CI 1.08–1.29), all-cause (1.29, 1.21–1.38), cardiovascular (1.43, 1.20–1.71), and non-cardiovascular mortality (1.28, 1.20–1.37; *p* < 0.001), while the risks of recurrent ischemic stroke/systemic thromboembolic events, ICH, and total bleeding events were not increased in patients with a greater renal function variability in the multivariate-adjusted analysis (all *p* > 0.05; Table 4).

#### 3.1.6 Sensitivity analysis

After excluding patients with baseline eGFR < 30 mL/min/1.73 m^2^, greater renal function variability was significantly associated with an increased risk of recurrent ischemic stroke and systemic embolism, total bleeding, MACE, all-cause, cardiovascular, and non-cardiovascular mortality (Supplementary Table 3). Similar findings were noted when we further excluded 816 patients who had an eGFR < 30 mL/min/1.73 m^2^ at any time point during the follow-up period (Supplementary Table 4). When using VIM and ARV as the indicators of renal function variability, we found greater renal function variability was also statistically significantly associated with various adverse clinical outcomes (Supplementary Tables 5, 6).

### 3.2 Hospital-based stroke registries

The clinical characteristics of patients from the hospital-based stroke registries are shown in Supplementary Table 7. A total of 804 patients were included in the final analysis after excluding patients who died within 90 days of the index event, had less than three renal function tests during the follow-up period, had underlying valvular heart disease/undergone valvular replacement, were on dialysis, or received both antiplatelet and anticoagulation treatment. The mean age was 78 ± 11 years, 48% were male, and the mean eGFR was 70 ± 22 mL/min/1.73 m^2^ at baseline. Out of the 804 patients, 581 (72%) were prescribed anticoagulants upon discharge, with 499 out of 581 (86%) being prescribed with DOACs. During a mean follow-up period of 2.66 ± 1.98 years (2,139 patient-years), patients with greater renal function variability were independently associated with an increased risk of ischemic stroke/systemic embolism, MACE, all-cause, cardiovascular, and non-cardiovascular mortality after adjusting for age, sex, and possible confounding factors (all p_trend_ < 0.05; Supplementary Figure 2; Supplementary Table 8).

## 4 Discussion

To the best of our knowledge, this is the first population-based study to investigate the prognostic implications of renal function variability in patients with TIA/ischemic stroke and AF. Independent of age, sex, baseline renal function, and vascular risk factors, higher renal function variability was independently associated with a number of adverse clinical outcomes, including ischemic stroke/systemic embolism, ICH, total bleeding, MACE, and mortality (cardiovascular and non-cardiovascular). These findings remained significant in the 1,738 patients who were prescribed DOACs on discharge. Similarly, higher renal function variability was independently associated with an increased risk of MACE and mortality (cardiovascular and non-cardiovascular) in patients prescribed with warfarin and antiplatelet agents upon discharge. However, higher renal function variability was not independently associated with the risk of recurrent ischemic stroke/systemic embolism, ICH, and total bleeding events in warfarin and antiplatelet users.

Our study found that patients with greater renal function variability were older and had more cardiovascular comorbidities and poorer renal function at baseline. The findings were coherent with previous studies (26). Patients with greater renal function variability were less likely to be prescribed with DOACs in our study. This may be due to the fact that DOACs rely on renal excretion at variable degrees, and they are contraindicated in patients with relatively advanced renal impairment (27). Furthermore, patients with greater renal function variability were more likely to have multiple comorbidities, rendering clinicians potentially more inclined to prescribe antiplatelet agents or no antithrombotic agents due to fear of bleeding complications.

In this study, we calculated three metrics (CV, VIM, and ARV), and the overall direction of effect was the same. The subdistribution hazard ratios decreased when using the ARV as the indicator of renal function variability. ARV averages the absolute differences in eGFR between consecutive visits (25), whereas CV and VIM capture the variation of all the eGFR measurements (28). Previous studies suggest that CV and VIM are highly correlated and may provide similar information (29). However, ARV captures variability from one visit to the next and may convey different information.

The overall mortality rate in our cohort is 170.5 events per 1,000 person-years (17.1% per year). A joint analysis of seven European and Japanese prospective cohorts of patients with AF following acute ischemic stroke found that the mortality rate for DOAC users was 6.3% per year and 10.8% per year for warfarin users (30). The aforementioned study excluded patients not on any anticoagulation treatment or patients who started anticoagulation treatment 90 days after the index stroke event. However, 43.4% of patients in our cohort were prescribed either only antiplatelet agents or no antithrombotic treatment. Patients not on adequate oral anticoagulation regimens were shown to have a higher mortality rate than those properly anticoagulated with either warfarin or DOACs (31). Additionally, the functional outcome would affect the mortality rate; however, neither study assessed the functional outcomes.

The results from our study show the value of renal function variability as a prognostic indicator beyond that of a single renal function measurement. Previous studies suggested that the variation in renal function is the result of both intrinsic and extrinsic factors. Intrinsic kidney diseases include renal microvascular disease, impairment of autoregulatory mechanisms, or limited renal function reserve while extrinsic factors include changes in intravascular volume status, congestive heart failure, liver disease, pulmonary disease, and/or use of medications that may influence renal function (11, 13). Renal function variability has been associated with an increased risk of all-cause mortality in patients with hypertension (12). Data from the TOPCAT trial also showed that increased renal function variability was associated with hospital admissions for heart failure and cardiovascular death in heart failure patients with preserved ejection fraction (14). For TIA/ischemic stroke patients with AF who need OACs for secondary stroke prevention, renal function variability is of utmost importance since all OACs depend on the kidney for clearance, albeit to varying degrees (6, 7). Patient’s exposure to OACs may be altered as a result of fluctuations in renal function and may lead to thrombotic or bleeding complications (32). Testa et al. found that plasma DOAC levels were associated with risks of recurrent ischemic events and bleeding complications (33, 34). Studies in Chinese patients showed that increasing plasma DOAC levels would increase the risk of bleeding complications in patients with renal impairment (32). It is therefore reasonable to postulate that fluctuations in renal function may reduce or increase plasma DOAC levels and thus contribute to a range of adverse clinical outcomes.

Previous studies have indicated that DOAC users experience a slower decline in renal function over time than the warfarin users, in both Asian (35) and Caucasian patients (36–38). A recent network meta-analysis also demonstrated that DOACs had a lower risk of bleeding complications and recurrent ischemic stroke/systemic thromboembolism than warfarin for patients with CrCl of ≥25 mL/min (39). However, we observed no significant differences in the renal function variability between patients prescribed with DOACs and warfarin (*p* = 0.155), nor in the proportions of DOAC and warfarin users who had a decline in the renal function during the follow-up period (*p* = 0.200).

The results from our study have several important clinical implications, which prompt further evaluation in future large-scale studies. Our study demonstrated that renal function variability over time holds important prognostic value and serves as an important risk factor in TIA/ischemic stroke patients with AF taking OACs. Regular monitoring of renal function and its changes over time is essential for the management of these patients, especially those with a lower baseline eGFR. With advances in technology and widely used electronic health record systems, renal function variability can be easily calculated and may provide a convenient tool to guide clinical management. While drug levels for various DOACs are now available in some centers and may likely vary depending on one’s renal function, especially if highly fluctuating, it is uncertain whether regular monitoring of DOAC drug levels with consequent adjustment of DOAC dosing has any benefit on clinical outcomes (40). Further studies in this area are required. Nevertheless, given that patients with high renal function variability are at increased risk of a range of adverse clinical events, patients with potential changes in renal function (e.g., volume depletion, new prescription of medications that may affect renal function, etc.) should be monitored closely and treated promptly to minimize renal function fluctuations.

Our study does have several limitations. First, our study was retrospective in nature, and thus, we cannot exclude the possibility of residual unmeasured confounding factors. Moreover, baseline risks of bleeding and ischemic events may be different among hospitalized patients and those managed in the outpatient setting. We were unfortunately unable to differentiate these patients in a population-based study. Furthermore, the frequency of renal function assessment was at the discretion of the attending physician, and it was not standardized. Therefore, the total number and the time interval of renal function tests for each individual were highly variable. Moreover, while the Cockcroft-Gault equation is recommended for the estimation of renal function in the context of DOAC dosing, due to the limitations of CDARS, we could only calculate the eGFR using the CKD-EPI equation (41). The number of patients prescribed warfarin in our cohort was small (*n* = 418); thus, our cohort will be underpowered to detect the associations of renal function variability with adverse clinical outcomes in patients on warfarin. Similarly, our study is also underpowered to determine the effects of renal function variability in patients taking individual DOACs. Furthermore, caution is warranted when generalizing the study findings, given that the majority of patients were Asian Chinese. Multiple studies have shown that DOACs are more effective and safer for Asian patients compared to Caucasian patients. The findings could be due to the fact that Asian patients had an increased risk of bleeding, and they had difficulties in maintaining the therapeutic range of the international normalized ratio (INR) of 2 to 3 when taking warfarin (42, 43). Moreover, studies also showed that a lower dose of DOACs was needed in Asian patients because of lower body mass index (44). Furthermore, our patients had a relatively high median number of renal tests. Thus, selection bias may be present in the study, as we included those patients who were more susceptible to a decline in renal function and thus may have had more inferior outcomes. Moreover, the exclusion of patients who survived less than 90 days may also introduce selection bias in the study. Inter-laboratory variation in serum creatinine measurement attributed to different calibration settings could also impact our results. The study did not take into account of speed of variability in renal function, which may also be important for patients’ outcomes. Finally, the condition of high renal function variability in patients due to their underlying acute renal impairment could not be identified from the analysis. Future large-scale prospective studies in patients of other ethnic groups will be needed to validate our findings.

## 5 Conclusion

Increased renal function variability is associated with adverse clinical outcomes in TIA/ischemic stroke patients with AF. Further large-scale studies are needed to validate our results.

## Data availability statement

The original contributions presented in the study are included in the article/Supplementary material, further inquiries can be directed to the corresponding author.

## Ethics statement

The studies involving humans were approved by the Institutional Review Board of the University of Hong Kong/Hospital Authority Hong Kong West Cluster. The studies were conducted in accordance with the local legislation and institutional requirements. Written informed consent for participation was not required from the participants or the participants’ legal guardians/next of kin in accordance with the national legislation and institutional requirements.

## Author contributions

XW: Writing – review & editing, Formal analysis, Conceptualization, Writing – original draft. C-fS: Writing – review & editing. K-CT: Writing – review & editing. WL: Writing – review & editing. Y-KW: Writing – review & editing, Data curation. RL: Writing – review & editing, Data curation. JF: Writing – review & editing. BI: Writing – review & editing. HK: Writing – review & editing. TL: Writing – review & editing. BS: Writing – review & editing. DY: Writing – review & editing. HL: Writing – review & editing. K-KL: Writing – review & editing, Supervision, Resources, Funding acquisition, Conceptualization. EY: Writing – review & editing.
