# Supplementary material for: Impact of renal function variability on long-term prognosis in ischemic stroke patients with atrial fibrillation
Source: Front Neurol. 2024 Apr 22;15:1294022. doi: 10.3389/fneur.2024.1294022 (PMC11071668; doi:10.3389/fneur.2024.1294022)
Supplement: Supplementary file 1 [file Data_Sheet_1.docx]

**Supplemental material**

**Impact of Renal function Variability on Long-Term Prognosis in Ischemic Stroke Patients with Atrial Fibrillation**

**Supplemental Table 1. International Classification of Diseases Codes, Ninth Revision, Clinical Modification (ICD-9-CM) used in the study**

**Supplemental Table 2. Competing risk regression analysis of renal function variability stratified by individual DOAC types**

**Supplemental Table 3. Competing risk regression analysis by renal function variability in patients with baseline eGFR ≥ 30 mL/min/1.73 m2**

**Supplemental Table 4. Competing risk regression analysis by renal function variability in patients with both baseline and follow-up eGFR ≥ 30 mL/min/1.73 m^2^**

**Supplemental Table 5. Competing risk regression analysis by eGFR-VIM**

**Supplemental Table 6. Competing risk regression analysis by eGFR-ARV**

**Supplemental Table 7. Clinical characteristics of patients within the hospital-based registry**

**Supplemental Table 8. Competing risk regression analysis by renal function variability of the hospital-based registry**

**Supplemental Figure 1. Adjusted restricted spline plots for risk of (A) recurrent ischemic stroke and systemic embolism, (B) intracerebral hemorrhage, (C) total bleeding, (D) major adverse cardiovascular events, (E) all-cause mortality, (F) cardiovascular mortality and (G) non-cardiovascular mortality over the range of eGFR CV among patients with TIA/ischemic stroke and AF.**

**Supplemental Figure 2. Risk of (A) recurrent ischemic stroke and systemic embolism, (B) intracerebral hemorrhage, (C) total bleeding, (D) major adverse cardiovascular events, (E) all-cause mortality, (F) cardiovascular mortality and (G) non-cardiovascular mortality among patients with TIA/ischemic stroke and atrial fibrillation from the hospital-based registry.**

**Supplemental Table 1. International Classification of Diseases Codes, Ninth Revision, Clinical Modification (ICD-9-CM) used in the study**

| **ICD-9-CM** | **Descriptions** |
| --- | --- |
| **Atrial fibrillation** |  |
| 427.3 | Atrial fibrillation and flutter |
|  |  |
| **Transient cerebral ischemia** | |
| 435 | Transient cerebral ischemia |
|  |  |
| **Ischemic stroke** | |
| 433 | Occlusion and stenosis of precerebral arteries |
| 434 | Occlusion of cerebral arteries |
| 436 | Acute, but ill-defined, cerebrovascular disease |
| 437 | Other and ill-defined cerebrovascular disease |
|  |  |
| **Valvular heart diseases/replacement** | |
| 394.0 | Mitral stenosis |
|  |  |
| **Valve replacement (procedure code)** | |
| 35.20 | Open and other replacement of unspecified heart valve |
| 35.22 | Open and other replacement of aortic valve |
| 35.24 | Open and other replacement of mitral valve |
| 35.26 | Open and other replacement of pulmonary valve |
| 35.28 | Open and other replacement of tricuspid valve |
| **Hypertension** |  |
| 401  402  403  404  405  **Diabetes** | Essential hypertension  Hypertensive heart disease  Hypertensive chronic kidney disease  Hypertensive heart and chronic kidney disease  Secondary hypertension |
| 250  **Hyperlipidemia** | Diabetes mellitus |
| 272 | Hyperlipidemia |
|  |  |
| **Ischemic heart disease/Coronary artery disease** | |
| 410 | Acute myocardial infarction |
| 411 | Other acute and subacute forms of ischemic heart disease |
| 412 | Old myocardial infarction |
| 413 | Angina pectoris |
| 414 | Other forms of chronic ischemic heart disease |
|  |  |
| **Congestive heart failure** | |
| 402.01 | Malignant hypertensive heart disease with congestive heart failure |
| 402.91 | Unspecified hypertensive heart disease with congestive heart failure |
| 404.01 | Hypertensive heart and renal disease, malignant, with congestive heart failure |
| 404.03 | Hypertensive heart and renal disease, malignant, with congestive heart failure and renal failure |
| 404.91 | Hypertensive heart and renal disease, unspecified, with congestive heart failure |
| 404.93 | Hypertensive heart and renal disease, unspecified, with congestive heart failure and renal failure |
| 428 | Heart failure |
|  |  |
| **Peripheral vascular disease** | |
| 443.9 | Peripheral vascular disease |
|  |  |
| **Systemic embolic events** | |
| 443 | Other peripheral vascular disease |
| 444 | Arterial embolism and thrombosis |
| 445 | Atheroembolism |
|  |  |
| **Intracranial hemorrhage** | |
| 430 | Subarachnoid hemorrhage |
| 431 | Intracerebral hemorrhage |
| 432.0 | Nontraumatic extradural hemorrhage |
| 432.1 | Subdural hemorrhage |
| 432.9 | Unspecified intracranial hemorrhage |
|  |  |
| **Gastrointestinal bleeding** | |
| 531 | Gastric ulcer |
| 532 | Duodenal ulcer |
| 533 | Peptic ulcer site unspecified |
| 534 | Gastrojejunal ulcer |
| 535 | Acute gastritis |
| 578 | Gastrointestinal hemorrhage |
|  |  |
| **Other gastrointestinal bleeding** | |
| 562.02 | Diverticulosis of small intestine with hemorrhage |
| 562.03 | Diverticulitis of small intestine with hemorrhage |
| 562.12 | Diverticulosis of colon with hemorrhage |
| 562.13 | Diverticulitis of colon with hemorrhage |
| 569.3 | Hemorrhage of rectum and anus |
| 569.85 | Angiodysplasia of intestine with hemorrhage |
| 569.86 | Dieulafoy lesion (hemorrhagic) of intestine |
| 792.4 | Nonspecific abnormal findings in saliva |
| 792.9 | Other nonspecific abnormal findings in body substances |
|  |  |
| **Other extracranial bleeding** | |
| 423.0 | Hemopericardium |
| 459.0 | Hemorrhage NOS |
| 593.81 | Vascular disorders of kidney |
| 599.7 | Hematuria |
| 623.8 | Other specified noninflammatory disorders of vagina |
| 626.2 | Excessive menstruation |
| 626.6 | Metrorrhagia |
| 719.1 | Hemarthrosis |
| 784.7 | Epistaxis |
| 784.8 | Hemorrhage from throat |
| 786.3 | Hemoptysis |
|  |  |
| **Dialysis** |  |
| 585.6 | End stage renal disease (requiring chronic dialysis) |
| 38.95 | Venous catheterization for renal dialysis |
| 39.27 | Arteriovenostomy for renal dialysis |
| 39.42 | Revision of arteriovenous shunt for renal dialysis |
| 39.43 | Removal of arteriovenous shunt for renal dialysis |
| 39.95 | Hemodialysis |
| 54.93 | Creation of cutaneoperitoneal fistula |
| 54.98 | Peritoneal dialysis (excludes peritoneal lavage) |
| 792.5 | Cloudy (hemodialysis)(peritoneal) dialysis effluent |
| 996.68 | Infection and inflammatory reaction due to vascular device, implant and graft (due to peritoneal dialysis catheter) |
| 996.73 | Other complications due to renal dialysis device, implant, and graft |
| V45.1 | Postsurgical renal dialysis status |
| V56 | Encounter for dialysis and dialysis catheter care |
| V56.0 | Extracorporeal dialysis |
| V56.1 | Fitting and adjustment of extracorporeal dialysis catheter |
| V56.2 | Fitting and adjustment of peritoneal dialysis catheter |
| V56.3 | Encounter for adequacy testing for dialysis |
| V56.8 | Other dialysis |

**Supplemental Table 2. Competing risk regression analysis of renal function variability stratified by individual DOAC types**

|  | **Renal function variability as an interval variable (per 1 SD increment in CV)** | | | | | |  |
| --- | --- | --- | --- | --- | --- | --- | --- |
|  | **Unadjusted SHR (95% CI)** | **P value** | **SHR (95% CI) adjusted for age and sex** | **P value** | **Multi-variate^[[1]](#footnote-1)^ adjusted SHR (95% CI)** | **P value** | **P for interaction** |
| **Ischemic stroke and systemic embolism** | | | | | | |  |
| Apixaban-users (n = 833) | 1.32 (1.10-1.58) | 0.003 | 1.38 (1.16-1.65) | <0.001 | 1.40 (1.16-1.67) | 0.005 | 0.906 |
| Dabigatran-users (n = 594) | 1.30 (1.03-1.64) | 0.029 | 1.35 (1.07-1.70) | 0.011 | 1.31 (1.04-1.66) | 0.024 |  |
| Rivaroxaban-users (n = 274) | 1.22 (0.98-1.52) | 0.074 | 1.23 (0.99-1.54) | 0.067 | 1.19 (0.94-1.51) | 0.140 |  |
| **Intracerebral hemorrhage** | | | | | | |  |
| Apixaban-users (n = 833) | 1.41 (1.08-1.84) | 0.011 | 1.40 (1.03-1.91) | 0.034 | 1.44 (1.09-1.90) | 0.010 | 0.905 |
| Dabigatran-users (n = 594) | 1.21 (0.84-1.76) | 0.310 | 1.11 (0.74-1.66) | 0.600 | 1.17 (0.78-1.75) | 0.450 |  |
| Rivaroxaban-users (n = 274) | 1.20 (0.73-1.98) | 0.460 | 1.19 (0.70-2.01) | 0.530 | 1.12 (0.70-1.78) | 0.640 |  |
| **Total bleeding** | | | | | | |  |
| Apixaban-users (n = 833) | 1.24 (1.10-1.40) | <0.001 | 1.23 (1.07-1.41) | 0.003 | 1.25 (1.09-1.43) | 0.001 | 0.102 |
| Dabigatran-users (n = 594) | 1.48 (1.29-1.69) | <0.001 | 1.46 (1.25-1.70) | <0.001 | 1.45 (1.24-1.70) | 0.001 |  |
| Rivaroxaban-users (n = 274) | 1.16 (0.91-1.48) | 0.220 | 1.09 (0.84-1.41) | 0.510 | 1.14 (0.88-1.49) | 0.330 |  |
| **Major adverse cardiovascular events** | | | | | | |  |
| Apixaban-users (n = 833) | 1.31 (1.20-1.44) | <0.001 | 1.29 (1.17-1.42) | <0.001 | 1.26 (1.13-1.40) | <0.001 | 0.202 |
| Dabigatran-users (n = 594) | 1.49 (1.31-1.70) | <0.001 | 1.44 (1.26-1.64) | <0.001 | 1.38 (1.19-1.59) | <0.001 |  |
| Rivaroxaban-users (n = 274) | 1.42 (1.21-1.67) | <0.001 | 1.39 (1.19-1.63) | <0.001 | 1.31 (1.11-1.54) | 0.001 |  |
| **All-cause mortality^[[2]](#footnote-2)^** | | | | | | |  |
| Apixaban-users (n = 833) | 1.75 (1.62-1.90) | <0.001 | 1.77 (1.63-1.93) | <0.001 | 1.81 (1.66-1.98) | <0.001 | <0.001 |
| Dabigatran-users (n = 594) | 2.21 (1.95-2.51) | <0.001 | 2.05 (1.79-2.35) | <0.001 | 2.13 (1.84-2.48) | <0.001 |  |
| Rivaroxaban-users (n = 274) | 2.37 (2.01-2.80) | <0.001 | 2.25 (1.89-2.68) | <0.001 | 2.39 (1.96-2.93) | <0.001 |  |
| **Cardiovascular mortality** |  |  |  |  |  |  |  |
| Apixaban-users (n = 833) | 1.87 (1.49-2.34) | <0.001 | 1.82 (1.47-2.25) | <0.001 | 1.87 (1.44-2.45) | <0.001 | 0.403 |
| Dabigatran-users (n = 594) | 1.23 (0.92-1.65) | 0.048 | 1.04 (0.72-1.51) | 0.820 | 1.07 (0.76-1.50) | 0.710 |  |
| Rivaroxaban-users (n = 274) | 2.81 (1.69-4.67) | <0.001 | 2.62 (1.55-4.42) | <0.001 | 2.42 (0.94-6.26) | 0.067 |  |
| **Non-cardiovascular mortality** |  |  |  |  |  |  |  |
| Apixaban-users (n = 833) | 1.74 (1.54-1.97) | <0.001 | 1.76 (1.60-1.95) | <0.001 | 1.81 (1.63-2.01) | <0.001 | 0.007 |
| Dabigatran-users (n = 594) | 2.30 (1.99-2.67) | <0.001 | 2.15 (1.84-2.51) | <0.001 | 2.24 (1.89-2.66) | <0.001 |  |
| Rivaroxaban-users (n = 274) | 2.34 (1.97-2.78) | <0.001 | 2.23 (1.86-2.66) | <0.001 | 2.39 (1.96-2.92) | <0.001 |  |

**Supplemental Table 3. Competing risk regression analysis by renal function variability in patients with baseline eGFR ≥ 30 mL/min/1.73 m^2^**

|  | **Renal functional variability as an interval variable (per 1 SD increment in CV)** | | | | | | | | | |
| --- | --- | --- | --- | --- | --- | --- | --- | --- | --- | --- |
|  | **Unadjusted SHR (95% CI)** | **P value** | | **SHR (95% CI) adjusted for age and sex** | | **P value** | | **Multi-variate^[[3]](#footnote-3)^ adjusted SHR (95% CI)** | **P value** | |
| **Ischemic stroke and systemic embolism** | 1.17 (1.09-1.26) | 0.001 | 1.17 (1.09-1.26) | | 0.001 | | 1.13 (1.04-1.22) | | | 0.003 |
| **Intracerebral hemorrhage** | 1.06 (0.91-1.24) | 0.440 | 1.08 (0.92-1.27) | | 0.320 | | 1.05 (0.90-1.23) | | | 0.540 |
| **Total bleeding** | 1.13 (1.06-1.20) | <0.001 | 1.11 (1.04-1.18) | | 0.002 | | 1.10 (1.03-1.18) | | | 0.004 |
| **Major adverse cardiovascular events** | 1.29 (1.21-1.37) | <0.001 | 1.27 (1.20-1.35) | | <0.001 | | 1.22 (1.15-1.30) | | | <0.001 |
| **All-cause mortality^[[4]](#footnote-4)^** | 1.52 (1.47-1.57) | <0.001 | 1.47 (1.42-1.52) | | <0.001 | | 1.43 (1.38-1.48) | | | <0.001 |
| **Cardiovascular mortality** | 1.54 (1.37-1.74) | <0.001 | 1.51 (1.35-1.70) | | <0.001 | | 1.47 (1.29-1.66) | | | <0.001 |
| **Non-cardiovascular mortality** | 1.52 (1.40-1.64) | <0.001 | 1.46 (1.38-1.55) | | <0.001 | | 1.42 (1.34-1.52) | | | <0.001 |

**Supplemental Table 4. Competing risk regression analysis by renal function variability in patients with both baseline and follow-up eGFR ≥ 30 mL/min/1.73 m^2^**

|  | **Renal function variability as an interval variable (per 1 SD increment in CV)** | | | | | |
| --- | --- | --- | --- | --- | --- | --- |
|  | **Unadjusted SHR (95% CI)** | **P value** | **SHR (95% CI) adjusted for age and sex** | **P value** | **Multi-variate^[[5]](#footnote-5)^ adjusted SHR (95% CI)** | **P value** |
| **Ischemic stroke and systemic embolism** | 1.24 (1.11-1.38) | <0.001 | 1.25 (1.12-1.39) | <0.001 | 1.21 (1.08-1.36) | 0.002 |
| **Intracerebral hemorrhage** | 1.04 (0.83-1.31) | 0.730 | 1.06 (0.83-1.34) | 0.650 | 1.06 (0.83-1.35) | 0.660 |
| **Total bleeding** | 1.12 (1.03-1.23) | 0.010 | 1.08 (0.98-1.19) | 0.120 | 1.13 (1.02-1.24) | 0.014 |
| **Major adverse cardiovascular events** | 1.31 (1.22-1.41) | <0.001 | 1.29 (1.20-1.38) | <0.001 | 1.27 (1.18-1.38) | <0.001 |
| **All-cause mortality^[[6]](#footnote-6)^** | 1.64 (1.54-1.73) | <0.001 | 1.48 (1.38-1.57) | <0.001 | 1.49 (1.39-1.60) | <0.001 |
| **Cardiovascular mortality** | 1.54 (1.30-1.83) | <0.001 | 1.43 (1.19-1.72) | <0.001 | 1.40 (1.14-1.71) | 0.002 |
| **Non-cardiovascular mortality** | 1.64 (1.54-1.75) | <0.001 | 1.48 (1.37-1.59) | <0.001 | 1.50 (1.39-1.62) | <0.001 |

**Supplemental Table 5. Competing risk regression analysis by eGFR-VIM**

|  | **Renal function variability as an interval variable (per 1 SD increment in VIM)** | | | | | |
| --- | --- | --- | --- | --- | --- | --- |
|  | **Unadjusted SHR (95% CI)** | **P value** | **SHR (95% CI) adjusted for age and sex** | **P value** | **Multi-variate^[[7]](#footnote-7)^ adjusted SHR (95% CI)** | **P value** |
| **Ischemic stroke and systemic embolism** | 1.25 (1.13-1.37) | <0.001 | 1.25 (1.14-1.38) | <0.001 | 1.20 (1.08-1.33) | <0.001 |
| **Intracerebral hemorrhage** | 1.28 (1.05-1.56) | 0.014 | 1.33 (1.09-1.64) | 0.006 | 1.26 (1.03-1.53) | 0.026 |
| **Total bleeding** | 1.21 (1.12-1.31) | <0.001 | 1.18 (1.09-1.29) | 0.001 | 1.22 (1.12-1.32) | <0.001 |
| **Major adverse cardiovascular events** | 1.52 (1.43-1.62) | <0.001 | 1.48 (1.39-1.58) | <0.001 | 1.42 (1.33-1.52) | <0.001 |
| **All-cause mortality^[[8]](#footnote-8)^** | 2.00 (1.89-2.11) | <0.001 | 1.80 (1.70-1.91) | <0.001 | 1.75 (1.65-1.86) | <0.001 |
| **Cardiovascular mortality** | 2.12 (1.77-2.54) | <0.001 | 1.98 (1.62-2.41) | <0.001 | 1.89 (1.52-2.35) | <0.001 |
| **Non-cardiovascular mortality** | 1.98 (1.87,2.10) | <0.001 | 1.78 (1.67,1.90) | <0.001 | 1.74 (1.62,1.86) | <0.001 |

**Supplemental Table 6. Competing risk regression analysis by eGFR-ARV**

|  | **Renal function variability as an interval variable (per 1 SD increment in ARV)** | | | | | |
| --- | --- | --- | --- | --- | --- | --- |
|  | **Unadjusted SHR (95% CI)** | **P value** | **SHR (95% CI) adjusted for age and sex** | **P value** | **Multi-variate^[[9]](#footnote-9)^ adjusted SHR (95% CI)** | **P value** |
| **Ischemic stroke and systemic embolism** | 1.11 (1.04-1.19) | 0.001 | 1.12 (1.04-1.19) | 0.001 | 1.08 (1.01-1.16) | 0.031 |
| **Intracerebral hemorrhage** | 0.95 (0.79-1.14) | 0.590 | 0.96 (0.80-1.16) | 0.690 | 0.98 (0.82-1.18) | 0.850 |
| **Total bleeding** | 1.11 (1.05-1.18) | <0.001 | 1.09 (1.03-1.16) | 0.005 | 1.09 (1.02-1.16) | 0.009 |
| **Major adverse cardiovascular events** | 1.30 (1.23-1.38) | <0.001 | 1.28 (1.21-1.36) | <0.001 | 1.24 (1.17-1.32) | <0.001 |
| **All-cause mortality^[[10]](#footnote-10)^** | 1.16 (1.10-1.22) | <0.001 | 1.18 (1.12-1.24) | <0.001 | 1.21 (1.15-1.28) | <0.001 |
| **Cardiovascular mortality** | 1.38 (1.18-1.61) | <0.001 | 1.38 (1.18-1.62) | <0.001 | 1.46 (1.26-1.70) | <0.001 |
| **Non-cardiovascular mortality** | 1.14 (1.07,1.20) | <0.001 | 1.15 (1.09,1.22) | <0.001 | 1.19 (1.12,1.26) | <0.001 |

**Supplemental Table 7. Clinical characteristics of patients within the hospital-based registry**

| **Baseline characteristics (N = 804)** |  |
| --- | --- |
| Age, y | 77.7 (11.3) |
| Male (%) | 383 (47.6) |
| Hypertension (%) | 596 (74.1) |
| Diabetes mellitus (%) | 223 (27.7) |
| Hyperlipidemia (%) | 301 (37.4) |
| Smokers (%) | 153 (19.0) |
| History of TIA/stroke (%) | 137 (17.0) |
| History of bleeding (%) | 65 (8.1) |
| History of ischemic heart disease (%) | 158 (19.7) |
| History of congestive heart failure (%) | 99 (12.3) |
| CHA_2_DS_2_–VASc score, median (IQR) | 4 (3-5) |
| HAS-BLED score, median (IQR) | 2 (1-6) |
| eGFR, ml/min/1.73m^2^ | 70.3 (21.8) |
| Alcohol consumption (%) | 107 (13.3) |
| **Index event** |  |
| TIA, n (%) | 132 (16.4) |
| Ischemic stroke, n (%) | 672 (83.6) |
| **Medication prescription** |  |
| Antiplatelet only (%) | 154 (19.2) |
| Anticoagulant only (%) | 581 (72.3) |
| Not on antithrombotic agents (%) | 69 (8.6) |
| ACEIs/ARBs (%) | 147 (18.3) |
| Statins (%) | 439 (54.6) |
| **Oral anticoagulant** |  |
| Warfarin (%) | 82 (10.2) |
| DOACs (%) | 499 (62.1) |
| **Follow up** |  |
| Mean follow up, years | 2.66 (1.98) |
| Patient follow-up year | 2139 |
| **Outcome** |  |
| Recurrent stroke (%) | 117 (14.6) |
| Ischemic stroke (%) | 96 (11.9) |
| Intracerebral hemorrhage (%) | 25 (3.1) |
| Systemic embolism (%) | 19 (2.4) |
| Major adverse cardiovascular event (%) | 234 (29.1) |
| Major extracranial bleeding, n (%) | 54 (6.7) |
| Gastrointestinal, n (%) | 42 (5.2) |
| Others, n (%) | 14 (1.7) |
| All-cause mortality (%) | 247 (30.7) |

Abbreviations: TIA = transient ischemic attack; IQR = interquartile range; eGFR = estimated glomerular ﬁltration rate; DOAC = direct oral anticoagulant; ACEIs = angiotensin-converting-enzyme inhibitors; ARBs = angiotensin receptor blockers;

**Supplemental Table 8. Competing risk regression analysis by renal function variability of the hospital-based registry**

| **Coefficient of variance of eGFR** | **Unadjusted SHR (95% CI)** | **P_trend_** | **SHR (95% CI) adjusted for age and sex** | **P_trend_** | **Multi-variate^[[11]](#footnote-11)^ adjusted SHR (95% CI)** | **P_trend_** |
| --- | --- | --- | --- | --- | --- | --- |
| **Ischemic stroke and systemic embolism** | | | | | | |
| Q1 | 1 | 0.002 | 1 | 0.002 | 1 | 0.002 |
| Q2 | 1.12 (0.58-2.16) |  | 1.14 (0.59-2.19) |  | 1.17 (0.61-2.24) |  |
| Q3 | 1.82 (1.00-3.30) |  | 1.88 (1.02-3.46) |  | 1.93 (1.05-3.55) |  |
| Q4 | 2.17 (1.21-3.87) |  | 2.24 (1.24-4.05) |  | 2.27 (1.25-4.09) |  |
| **Intracerebral hemorrhage** | | | | | | |
| Q1 | 1 | 0.520 | 1 | 0.450 | 1 | 0.450 |
| Q2 | 1.54 (0.45-5.24) |  | 1.58 (0.46-5.40) |  | 1.60 (0.47-5.45) |  |
| Q3 | 1.26 (0.35-4.51) |  | 1.34 (0.37-4.81) |  | 1.34 (0.38-4.72) |  |
| Q4 | 1.64 (0.49-5.50) |  | 1.75 (0.54-5.67) |  | 1.73 (0.54-5.58) |  |
| **Major Bleeding** | | | | | | |
| Q1 | 1 | 0. 016 | 1 | 0.120 | 1 | 0.130 |
| Q2 | 1.19 (0.56-2.50) |  | 1.08 (0.51-2.30) |  | 1.05 (0.50-2.23) |  |
| Q3 | 1.44 (0.70-2.95) |  | 1.13 (0.55-2.34) |  | 1.10 (0.53-2.27) |  |
| Q4 | 2.15 (1.10-4.20) |  | 1.63 (0.83-3.20) |  | 1.61 (0.82-3.16) |  |
| **Major adverse cardiovascular events** | | | | | | |
| Q1 | 1 | <0.001 | 1 | <0.001 | 1 | <0.001 |
| Q2 | 1.35 (0.84-2.17) |  | 1.35 (0.84-2.17) |  | 1.31 (0.80-2.13) |  |
| Q3 | 2.37 (1.54-3.67) |  | 2.31 (1.48-3.59) |  | 2.14 (1.34-3.41) |  |
| Q4 | 2.96 (1.94-4.51) |  | 2.88 (1.87-4.44) |  | 2.69 (1.69-4.27) |  |
| **All-cause mortality**^[[12]](#footnote-12)^ | | | | | | |
| Q1 | 1 | <0.001 | 1 | <0.001 | 1 | <0.001 |
| Q2 | 1.16 (0.66-2.04) |  | 0.93 (0.53-1.62) |  | 0.96 (0.54-1.71) |  |
| Q3 | 2.99 (1.84-4.87) |  | 1.81 (1.10-2.96) |  | 1.91 (1.15-3.19) |  |
| Q4 | 5.90 (3.71-9.37) |  | 3.54 (2.21-5.66) |  | 3.57 (2.18-5.86) |  |
| **Cardiovascular mortality** |  |  |  |  |  |  |
| Q1 | 1 | <0.001 | 1 | 0.013 | 1 | 0.038 |
| Q2 | 0.59 (0.19-1.85) |  | 0.48 (0.16-1.47) |  | 0.46 (0.15-1.42) |  |
| Q3 | 3.29 (1.44-7.53) |  | 2.07 (0.87-4.91) |  | 1.96 (0.84-4.60) |  |
| Q4 | 3.08 (1.32-7.21) |  | 1.93 (0.80-4.66) |  | 1.61 (0.63-4.09) |  |
| **Non-cardiovascular mortality** | | | | | | |
| Q1 | 1 | <0.001 | 1 | <0.001 | 1 | <0.001 |
| Q2 | 1.56 (0.79-3.09) |  | 1.24 (0.62-2.45) |  | 1.31 (0.66-2.63) |  |
| Q3 | 3.02 (1.62-5.64) |  | 1.80 (0.95-3.42) |  | 1.98 (1.04-3.77) |  |
| Q4 | 7.61 (4.25-13.61) |  | 4.48 (2.48-8.11) |  | 4.66 (2.52-8.62) |  |

**
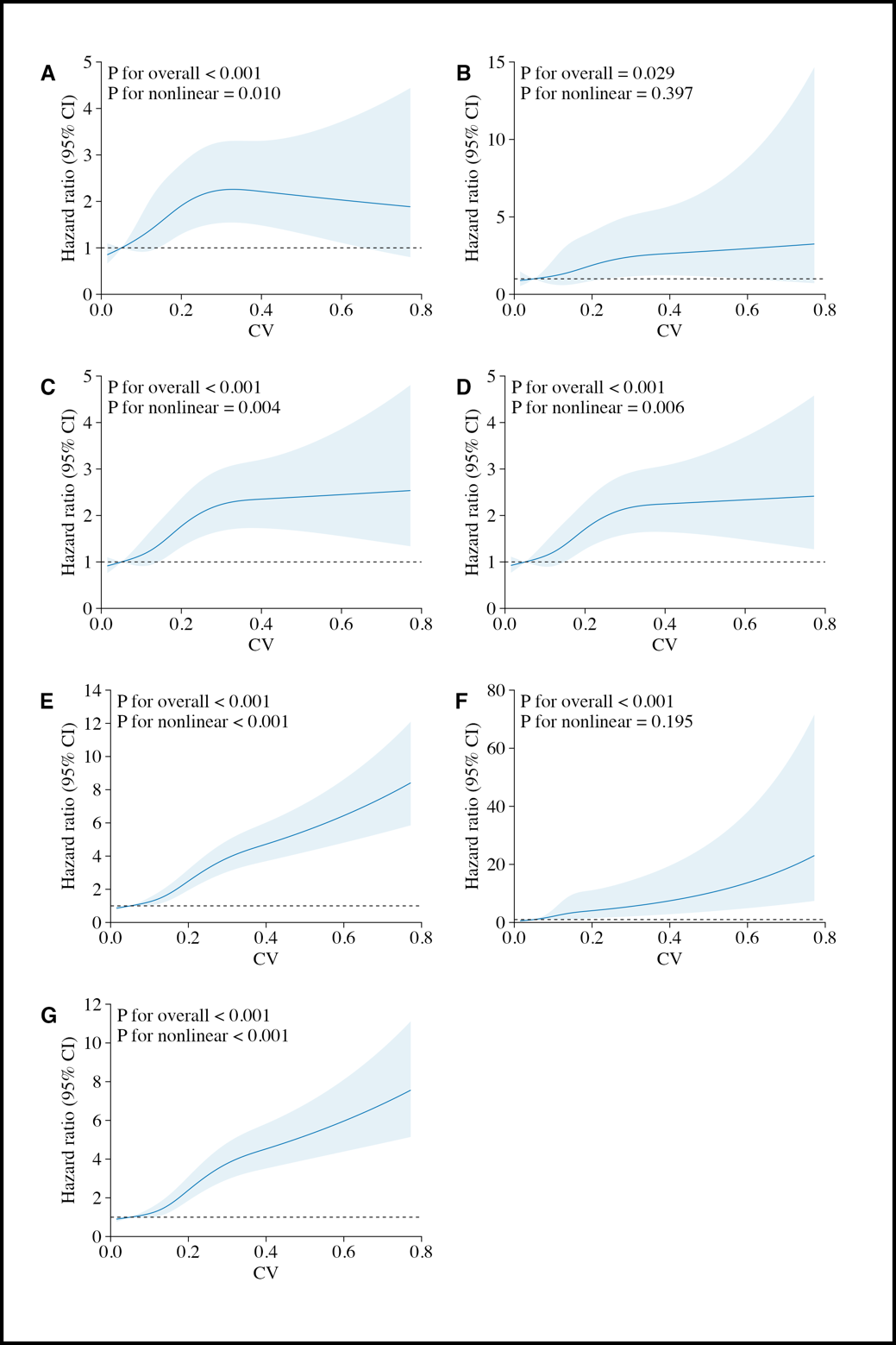
**

**Supplemental Figure 1. Adjusted restricted spline plots for risk of (A) recurrent ischemic stroke and systemic embolism, (B) intracerebral hemorrhage, (C) total bleeding, (D) major adverse cardiovascular events, (E) all-cause mortality, (F) cardiovascular mortality and (G) non-cardiovascular mortality over the range of eGFR CV among patients with TIA/ischemic stroke and AF.** **Co-variates included age, sex, hypertension, hyperlipidemia, diabetes mellitus, history of TIA/stroke, history of ischemic heart disease, eGFR at baseline, antithrombotic treatment, angiotensin converting enzyme inhibitor, angiotensin receptor blocker and statin-use. Co-variates with a p value <0.1 in the univariate model were included in the final model.** **Abbreviations: CV = coefficient of variation; CI = confidence interval; eGFR = estimated glomerular ﬁltration rate; TIA = transient ischemic attack.**


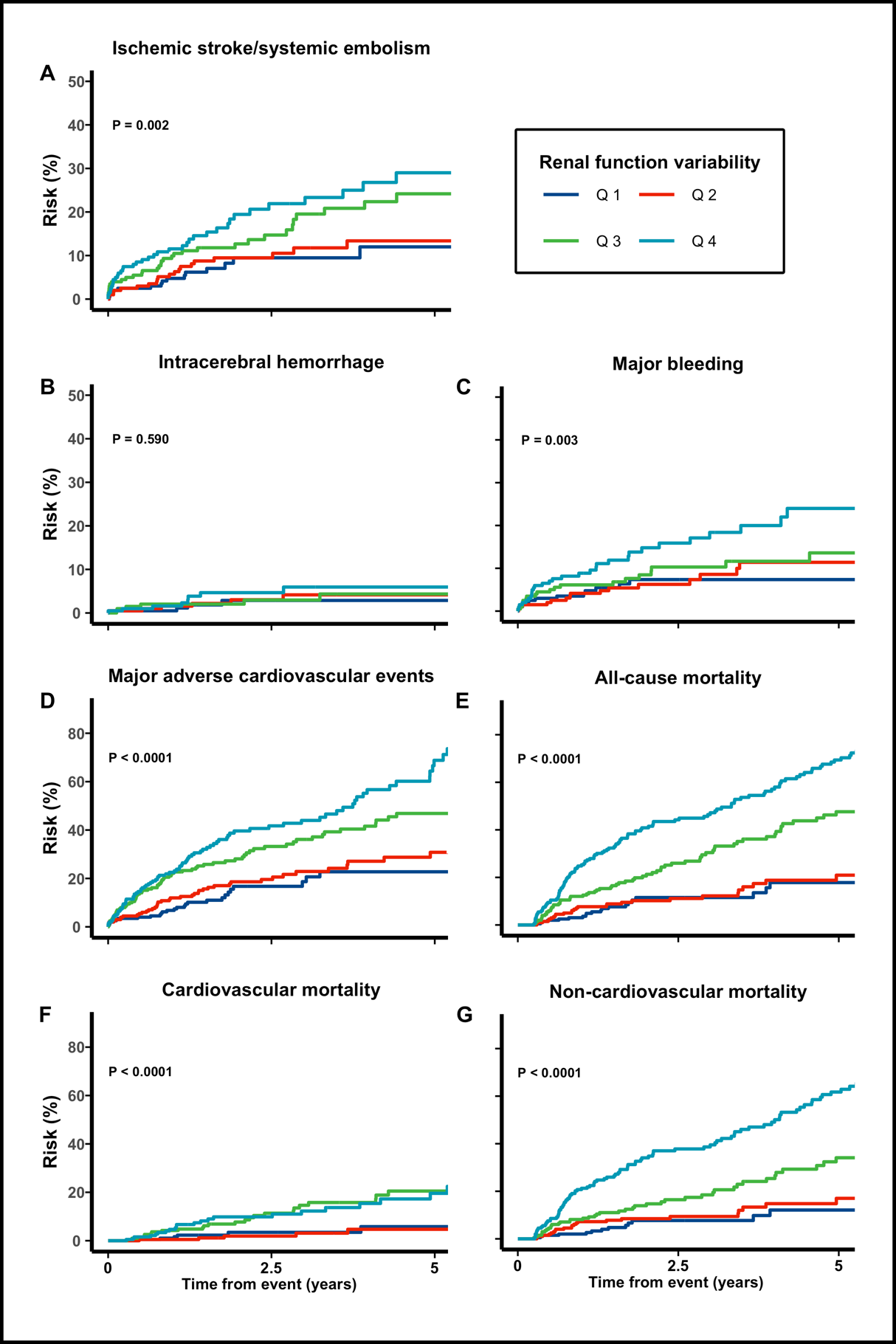


**Supplemental Figure 2.** **Risk of (A) recurrent ischemic stroke and systemic embolism, (B) intracerebral hemorrhage, (C) total bleeding, (D) major adverse cardiovascular events, (E) all-cause mortality, (F) cardiovascular mortality and (G) non-cardiovascular mortality among patients with TIA/ischemic stroke and atrial fibrillation from the hospital-based registry.**

1. Co-variates included age, sex, hypertension, hyperlipidemia, diabetes mellitus, history of TIA/stroke, history of ischemic heart disease, eGFR at baseline, angiotensin converting enzyme inhibitor, angiotensin receptor blocker and statin-use. Co-variates with a p value <0.1 in the univariate model were included in the final model. [↑](#footnote-ref-1)
2. Hazard ratio

   Abbreviations: SD = standard deviation; CV = coefficient of variation; SHR = subdistribution hazard ratio; CI = confidence interval; eGFR = estimated glomerular ﬁltration rate; TIA = transient ischemic attack; DOAC = direct oral anticoagulants. [↑](#footnote-ref-2)
3. Co-variates included age, sex, hypertension, hyperlipidemia, diabetes mellitus, history of TIA/stroke, history of ischemic heart disease, eGFR at baseline, antithrombotic treatment, angiotensin converting enzyme inhibitor, angiotensin receptor blocker and statin-use. Co-variates with a p value <0.1 in the univariate model were included in the final model. [↑](#footnote-ref-3)
4. Hazard ratios

   Abbreviations: SD = standard deviation; CV = coefficient of variation; SHR = subdistribution hazard ratio; CI = confidence interval; eGFR = estimated glomerular ﬁltration rate; TIA = transient ischemic attack; [↑](#footnote-ref-4)
5. Co-variates included age, sex, hypertension, hyperlipidemia, diabetes mellitus, history of TIA/stroke, history of ischemic heart disease, eGFR at baseline, antithrombotic treatment, angiotensin converting enzyme inhibitor, angiotensin receptor blocker and statin-use. Co-variates with a p value <0.1 in the univariate model were included in the final model. [↑](#footnote-ref-5)
6. Hazard ratios

   Abbreviations: SD = standard deviation; CV = coefficient of variation; SHR = subdistribution hazard ratio; CI = confidence interval; eGFR = estimated glomerular ﬁltration rate; TIA = transient ischemic attack; [↑](#footnote-ref-6)
7. Co-variates included age, sex, hypertension, hyperlipidemia, diabetes mellitus, history of TIA/stroke, history of ischemic heart disease, eGFR at baseline, antithrombotic treatment, angiotensin converting enzyme inhibitor, angiotensin receptor blocker and statin-use. Co-variates with a p value <0.1 in the univariate model were included in the final model. [↑](#footnote-ref-7)
8. Hazard ratio

   Abbreviations: SD = standard deviation; VIM = variability independent of mean; SHR = subdistribution hazard ratio; CI = confidence interval; eGFR = estimated glomerular ﬁltration rate; TIA = transient ischemic attack. [↑](#footnote-ref-8)
9. Co-variates included age, sex, hypertension, hyperlipidemia, diabetes mellitus, history of TIA/stroke, history of ischemic heart disease, eGFR at baseline, antithrombotic treatment, angiotensin converting enzyme inhibitor, angiotensin receptor blocker and statin-use. Co-variates with a p value <0.1 in the univariate model were included in the final model. [↑](#footnote-ref-9)
10. Hazard ratio

    Abbreviations: ARV = average real variability; SD = standard deviation; SHR = subdistribution hazard ratio; CI = confidence interval; eGFR = estimated glomerular ﬁltration rate; TIA = transient ischemic attack. [↑](#footnote-ref-10)
11. Co-variates included age, sex, hypertension, dyslipidemia, DM, history of CHF, history of IHD, history of TIA/stroke, history of bleeding, eGFR at baseline, alcohol consumption, antithrombotic treatment, ACEIs, ARBs, statins. Co-variates with a p value <0.1 in the univariate model were included in the final model; [↑](#footnote-ref-11)
12. Hazard ratio

    Abbreviations: SHR = subdistribution hazard ratio; CI = confidence interval; DM = diabetes mellitus; CHF = congestive heart failure; IHD = ischemic heart disease; eGFR = estimated glomerular ﬁltration rate; TIA = transient ischemic attack; ACEIs = angiotensin-converting-enzyme inhibitors; ARBs = angiotensin receptor blockers; [↑](#footnote-ref-12)
